# Asthma Attacks in Children—Challenges and Opportunities

**DOI:** 10.1007/s12098-021-04069-w

**Published:** 2022-01-21

**Authors:** Helena Jones, Adam Lawton, Atul Gupta

**Affiliations:** 1 Department of Pediatrics, North Middlesex Hospital, London, UK; 2 Pediatric Respiratory Department, King’s College Hospital, Denmark Hill, Brixton, London, SE5 9RS UK

**Keywords:** Asthma, Children, Respiratory, Pulmonology, Asthma attack, Asthma exacerbation, Acute asthma

## Abstract

Asthma is the most common chronic disease of childhood worldwide, and is responsible for significant morbidity and mortality in children and young people (CYP). Given the inherent dangers of a child experiencing even a single asthma attack, it is essential to identify and manage modifiable risk factors at every clinical opportunity. Following an attack, there is an opportunity to prevent future attacks by assessing compliance and optimizing asthma control. Careful questioning will allow physicians to identify asthma triggers, barriers to good asthma control, and health beliefs or socioeconomic obstacles that may have contributed to this attack. The vast majority of children with asthma can achieve good symptom control with appropriate use of low-dose inhaled corticosteroids.

**Supplementary Information:**

The online version contains supplementary material available at 10.1007/s12098-021-04069-w.

## Introduction

Asthma is the most common chronic disease of childhood worldwide, and is responsible for significant morbidity and mortality in children and young people (CYP). Asthma results from interactions between genetic and environmental factors that ultimately lead to airway hyper-reactivity, bronchial inflammation, and airway remodelling. This manifests clinically as variable airflow obstruction and respiratory symptoms such as wheeze, cough, chest tightness, and breathlessness.

Poorly-controlled asthma is associated with persistent symptoms, accelerated decline in lung function, and a greatly increased risk of future attacks, which may be life-threatening. The majority of CYP with asthma can achieve good symptom control through regular use of inhaled corticosteroids (ICS). Despite the dawn of new biologic controller therapies, access to these treatments is limited due to relatively high costs. However, most patients will never require these therapies, and only a minority of those with asthma have true therapy-resistant severe asthma [1]. Poor asthma control is more likely the result of difficulties with medication adherence, lack of asthma education, management of comorbidities, and social and environmental factors.

## Definition of Asthma Attacks

The phrase ‘asthma attack’ or ‘lung attack’ has been proposed as a preferable term to ‘asthma exacerbation’, reflecting the severity of an incident that is not merely a temporary inconvenience but a potentially fatal event that may cause lasting and progressive lung injury [2]. Others use the term ‘flare-up’, which implies that asthma is a chronic disease that remains present even when the patient is not symptomatic [3].

An asthma attack represents a sudden decrease in lung function (measurable by a fall in forced expiratory volume in 1 s (FEV1) and peak expiratory flow rate (PEFR)), accompanied by respiratory symptoms. Objective measurement of PEFR is used in the stratification of asthma attack severity in children, although it must be noted that this can be difficult in those who are severely unwell, or very young [4]. For the purposes of directing emergency treatment of an asthma attack, many asthma guidelines stratify the severity of the attack according to presenting features including PEFR.

## Impact of Asthma Attacks

Asthma attacks frequently require hospital admissions, and consequently children with poorly controlled asthma may suffer the multiplying effects of poor sleep due to nocturnal symptoms, poor school attendance due to hospital admissions, and reduced participation in recreational sports due to fears of worsening symptoms. The impact of frequent asthma attacks is also felt on the wider family, with parents having to miss work and lose income to care for their children during hospital admissions [5].

## Modifiable Risk Factors for Asthma Attacks

Given the inherent dangers of a child experiencing even a single asthma attack, it is essential to identify and manage modifiable risk factors at every clinical opportunity. Risk factors for asthma attacks include genetic, environmental, socioeconomic, and clinical management factors.

Current level of asthma control is a risk factor for future asthma attacks, as is recent need for a course of oral steroids or hospitalization [6]. Over-reliance on short-acting beta-agonist medications (SABA), inadequate ICS [7], and poor inhaler technique [8] all contribute to poor asthma control. Comorbidities such as rhinitis, gastro-oesophageal reflux, dysfunctional breathing, anxiety, psychological issues, obstructive sleep apnea, and obesity exacerbate symptoms if not optimally managed. Elevated fractional exhaled nitric oxide (FeNO) [9], low FEV1 [10], high bronchodilator reversibility [11], and higher levels of sputum eosinophilia [12] have all been associated with a higher likelihood of asthma exacerbations. Environmental risk factors include exposure to household allergens, poor air quality [13] and exposure to respiratory viruses.

Children from socially disadvantaged families have worse asthma control and are at increased risk of asthma attacks for a number of reasons including increased likelihood of exposure to allergens such as tobacco smoke, both indoor and outdoor air pollution in areas of low socioeconomic housing developments, and poorer financial and educational status impacting ability to access healthcare facilities and medication [14]. Psychological stress and exposure to negative life events also increase risk of asthma attacks, and the direct effect of psychosocial stressors on lung inflammation has been observed [15]. Education has been shown to reduce the frequency of acute hospital presentations [16], as well as improve the quality of life and reduce mortality in CYP with asthma [17]. CYP from certain minority ethnic groups have poorer asthma outcomes in the United Kingdom, and increasing scrutiny is being paid to the operational barriers that prevent children with ancestral origins in India, Pakistan, and Bangladesh from accessing asthma care [18].

## After an Asthma Attack: An Opportunity for Intervention

Following an attack, there is an opportunity to prevent future attacks by assessing compliance and optimizing asthma control. Careful questioning will allow physicians to identify asthma triggers, barriers to good asthma control, and health beliefs or socioeconomic obstacles that may have contributed to this attack.

When enquiring about compliance, it is important to adopt a nonaccusatory approach. One suggested way of sensitively enquiring without sounding accusatory is as follows; “many patients don’t use their inhalers as prescribed. In the last four weeks, how many days a week have you been taking it—not at all, 1 day a week, 2, 3 or more?” [19].

Provided inhaler technique and compliance have been optimzed and modifiable risk factors addressed, the clinician may consider stepping-up treatment. After any step-up in treatment, symptoms should be reviewed 2–3 mo later. If there has been no response, treatment should be stepped back down to the previous level, and alternative management options, or referral to a specialist respiratory pediatrician, should be considered.

## Confirming the Diagnosis of Asthma

As the symptoms of an asthma attack can sometimes be difficult to differentiate from symptoms of other respiratory diseases in children, it is sensible to ensure that asthma is the correct diagnosis (Supplementary Box S1).

The clinical history should be revisited in full to ensure that an alternative diagnosis or comorbidity has not been overlooked, and the child should undergo a clinical examination. Clinical examination may be normal even in the presence of asthma, especially if examined when not symptomatic. Although wheeze is one of the cardinal respiratory symptoms of asthma, there is significant discrepancy between what ‘wheeze’ means to parents and physicians [20]. Repeated normal examination of the chest when symptomatic does, however, reduce the probability of asthma.

There is no consensus regarding the diagnostic requirements for asthma in children, and previously the diagnosis has been made based on a thorough history and clinical examination [21]. The European Respiratory Society (ERS) task force on asthma has recently published evidence-based guidelines on diagnosing asthma in children [22]. The ERS guidelines recommend against making a diagnosis on clinical grounds, which was advocated in the BTS/SIGN guidelines [4]; or by using PEFR variability, which is an acceptable diagnostic test in the Global Initiative for Asthma (GINA) guidelines [19]. ERS guidelines propose a diagnostic algorithm for symptomatic children aged 5–16. In this algorithm, the simplest diagnostic pathway is by identifying abnormal spirometry and then subsequently demonstrating significant bronchodilator reversibility (Supplementary Fig. S1). In low-resource settings, such as developing countries, spirometry may not always be possible and hence, using gold-standard methods is not attainable. In this instance, the clinician may choose to follow guidance from GINA which recommends, in the absence of spirometry, providing the patient with a PEFR diary to assess diurnal PEFR variability (requiring only a basic PEFR meter) from twice-daily readings over 1 to 2 wk. In CYP, diurnal variation greater than 13% is considered excessive. If PEFR is also not possible, GINA recommends a decision whether to start asthma treatment should depend on clinical urgency and availability to other testing elsewhere [19].

## Assessing Asthma Control

There are several validated questionnaires for assessing asthma control in CYP, including the Asthma Control Questionnaire (ACQ) [23], Childhood Asthma Control Test (C-ACT) [24], Pediatric Asthma Quality-of-life Questionnaire (PAQLQ) [25], Test for Respiratory and Asthma Control in Kids (TRACK) [26], and Composite Asthma Severity Index (CASI) [27].

There is no data regarding which of these questionnaires, if any, is superior for assessing asthma control in a real-world setting. However, using a validated questionnaire for assessing asthma control is strongly recommended; the most commonly used are ACQ and C-ACT.

## Management of an Acute Asthma Attack

The treatment aims during an acute asthma attack are to prevent respiratory failure and treat respiratory symptoms and acute respiratory distress by reversing bronchoconstriction and reducing lung inflammation. The fundamental pharmacological management of acute asthma includes the use of bronchodilators, oxygen, and steroids.

Oxygen should be considered a prescribed medication and its use titrated to achieve targeted oxygen saturations of 94%–98%. Whilst it is clearly essential to avoid hypoxemia and consequent organ damage, hyperoxemia has no clinical benefit and has been shown to be harmful in a range of circumstances—likely stemming from oxygen’s role as a free radical producing molecule. Furthermore, evidence from adult data suggests that oxygen does not reduce the sensation of breathlessness in nonhypoxaemic patients, obviating its use for this indication in those not already hypoxemic [28].

Bronchoconstriction in acute asthma is chiefly combated with inhaled SABA (such as salbutamol), delivered every 20 min for one hour via an inhaler device with a spacer. A Cochrane review on additional bronchodilator use in children with acute asthma found that anticholinergic medication such as ipratropium bromide reduces the side effects of nausea and tremor, commonly caused by heavy salbutamol use during an acute attack. Both ipratropium and intravenous magnesium sulfate were found to reduce the risk of hospital admission, but no additional medication was found to reduce the risk of admission to intensive care [29].

Steroids are essential in acute asthma to combat the underlying lung inflammation. They work by binding to cellular glucocorticoid receptors, leading to the down-regulation of transcription of various proinflammatory molecules. Provided a patient can tolerate oral medication, there is no benefit of giving these medications intravenously or intramuscularly. Oral steroids take several hours to have an effect, so should be administered promptly in the Emergency Department. If given within one hour (‘The Golden Hour’) of arriving in the Emergency Department, the need for hospital admission is significantly reduced [30]. A Cochrane review was unable to demonstrate superiority of any particular steroid due to paucity of high-quality evidence [31].

The 2021 GINA guidelines [19] recommend against antibiotics and obtaining a chest radiograph in most children with an asthma attack requiring treatment in the Emergency Department.

## Preventing Asthma Attacks: Pharmacological Management

The vast majority of CYP with asthma can achieve good symptom control with appropriate use of low-dose ICS. Traditionally, pharmacological management in children has followed a stepwise approach that begins regular preventer therapy with inhaled corticosteroids (Supplementary Fig. S2). In the face of ongoing asthma symptoms, increased doses of ICS or additional controller therapies are trialled before referral for specialist management and the consideration of regular oral corticosteroids, or the newer biologic drugs such as omalizumab and mepolizumab. These biologic agents are monoclonal antibodies targeted at various components of the inflammatory pathways that cause asthma. For selected patients, biologics can lead to improved symptom control, fewer asthma attacks, and reduced use of oral steroids [32].

Recently, attention has focused on the safety of only prescribing intermittent SABA reliever therapy, following evidence that many children who subsequently die from an asthma attack are seen to have used these medications heavily, and have been underprescribed the essential steroid medications that would control the inflammation causing their asthma. The 2014 National Review of Asthma Deaths (NRAD) in the UK found that 9% of deaths were in those using SABA only; and of these, 39% had received excess prescriptions for SABA, indicating over-reliance on SABA without adequate control of the underlying mechanisms of disease [33].

It is crucial that asthma treatment is targeted at the underlying pathophysiological mechanisms of chronic inflammation. Therefore, the 2021 GINA guidelines have proposed that children should be started on a combined short-acting beta-agonist with low-dose inhaled corticosteroid inhaler, ensuring that a dose of steroid is received every time a child requires reliever treatment for asthma symptoms [19].

## Preventing Asthma Attacks: Nonpharmacological Management

Despite the plethora of novel or advanced treatments, it must be re-emphasised that asthma attacks in children and young people can be significantly reduced by identifying and treating modifiable risk factors (Supplementary Box S2).

Asthma education should cover topics that include correct use of inhaler devices with spacers, adherence to controller therapy, recognizing asthma attacks and when asthma control is poor, the importance of exercise and diet, and the treatment of modifiable risk factors in the personal environment.

All children with asthma should be provided with a written asthma action plan that explains how to identify deteriorating asthma control and how to respond. Evidence has shown that the provision of personalized asthma action plans improve caregiver's self-confidence and reduces the recurrence of asthma symptoms at 14 d following an Emergency Department presentation [34]. Lack of a personalized asthma action plan was identified in the 2014 NRAD report as a risk factor for a fatal asthma attack. GINA provides a sample plan in their asthma toolbox (Supplementary Fig. S3) [35].

## Future Directions of Research

Asthma is increasingly being considered an umbrella term for a group of respiratory diseases of varied etiology that have distinct pathophysiological mechanisms which lead to similar symptoms [36]. Low-cost biomarkers that can identify specific asthma phenotypes are being actively sought, with the aim of categorizing people with asthma into cohorts likely to respond to specific novel therapies [37]. As the cost of the novel biologics fall, more patients are likely to benefit from access to these effective, steroid-sparing agents.

The COVID-19 pandemic has accelerated technological innovation in healthcare. Home monitoring of lung function, home self-administration of biologic medication, and remote consultations have all taken place in the UK recently. Remote physical examination is also now possible with devices that enable a clinician to remotely auscultate the lungs, and more [38]. These innovations suggest that remote consultations for asthma management may well become more popular in the near future.

## Supplementary Information

Below is the link to the electronic supplementary material.

Supplementary file1 (DOCX 2512 KB)
