# Supplementary material for: Asthma Attacks in Children—Challenges and Opportunities
Source: Indian J Pediatr. 2022 Jan 21;89(4):373–7. doi: 10.1007/s12098-021-04069-w (PMC8776553; doi:10.1007/s12098-021-04069-w)
Supplement: Supplementary file 1 — Supplementary file1 (DOCX 2512 KB) [file 12098_2021_4069_MOESM1_ESM.docx]

**Supplementary Box S1** When should I consider an alternative diagnosis to asthma

(in CYP < 5 y?)

| The Global Initiative for Asthma (GINA) 2021 Report suggests that in a child 5 years or younger, any of the following features suggest an alternative diagnosis and indicate the need for further investigations:   - Failure to thrive - Neonatal or very early onset of symptoms (especially if associated with failure to thrive) - Vomiting associated with respiratory symptoms - Continuous wheezing - Failure to respond to asthma medications (inhaled ICS, oral steroids or SABA) - No association of symptoms with typical triggers, such as viral URTI - Focal lung or cardiovascular signs, or finger clubbing - Hypoxemia outside context of viral illness |
| --- |

**Supplementary Box S2** Top tips for managing CYP with frequent asthma attacks

| - Confirm that the primary diagnosis is indeed asthma - If poor control, consider underlying comorbidities that complicate asthma and make its treatment more difficult. - At every review, check the three Cs of asthma care:   - Control: are asthma symptoms well-controlled?   - Compliance: is poor-compliance a cause of poor control?   - Co-morbidities: are treatable co-morbidities present? - Consider starting all patients on a combination low-dose ICS/SABA inhaler, as per the latest GINA guidelines - Remember that poor asthma control is more likely related to socio-economic barriers for example increased exposure to allergens including tobacco smoke, poorer compliance with management, and reduced ability to access healthcare and obtain medications than sub-optimal physiological responses to treatment - how can these barriers be addressed in your practice? - Personalised self-management asthma action plans have been shown to improve asthma control - does your team provide these plans to all children? - Making asthma education a priority - Passive and active smoking cessation should be discussed at every clinic visit - Exploring barriers to good asthma control – are they taking treatment, confirm that inhaler technique is appropriate, revisit the asthma action plan, explore any concerns about the adverse effects of treatment |
| --- |


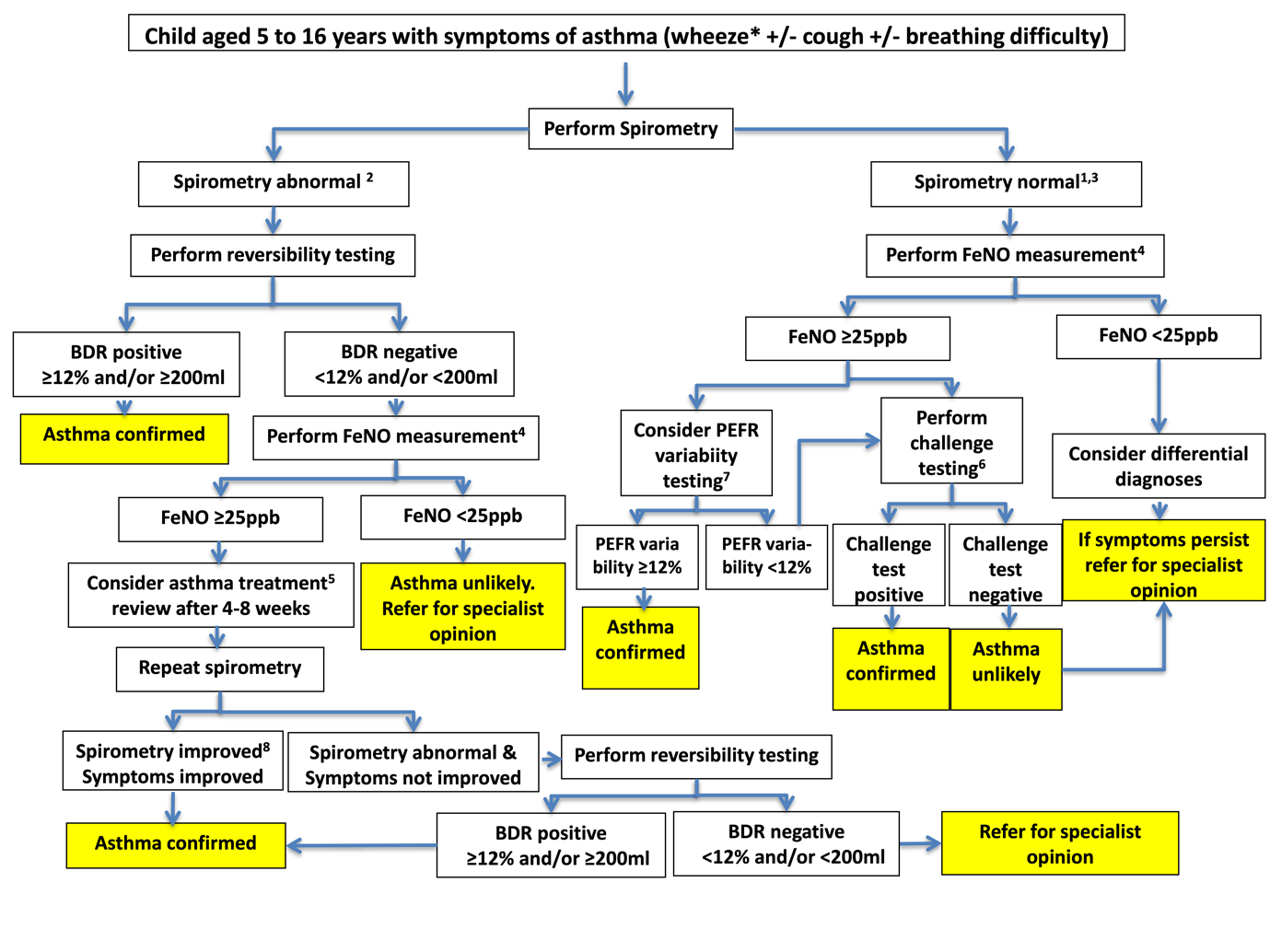


**Supplementary Fig. S1** ERS diagnostic algorithm for asthma in CYP 5–16 y

Reproduced, with permission, from: European Respiratory Society clinical practice guidelines for the diagnosis of asthma in children aged 5–16 years.Erol A. Gaillard, Claudia E. Kuehni, Steve Turner, MyroforaGoutaki, Karl A. Holden, Carmen C.M. de Jong, Christiane Lex, David K.H. Lo, Jane S. Lucas, Fabio Midulla, Rebeca Mozun, Giorgio Piacentini, David Rigau , Bart Rottier, Mike Thomas, Thomy Tonia, Jakob Usemann, Ozge Yilmaz, Angela Zacharasiewicz, Alexander Moeller. European Respiratory Journal (In Press) 2004173; DOI: 10.1183/13993003.04173-2020 Published 19 April 2021


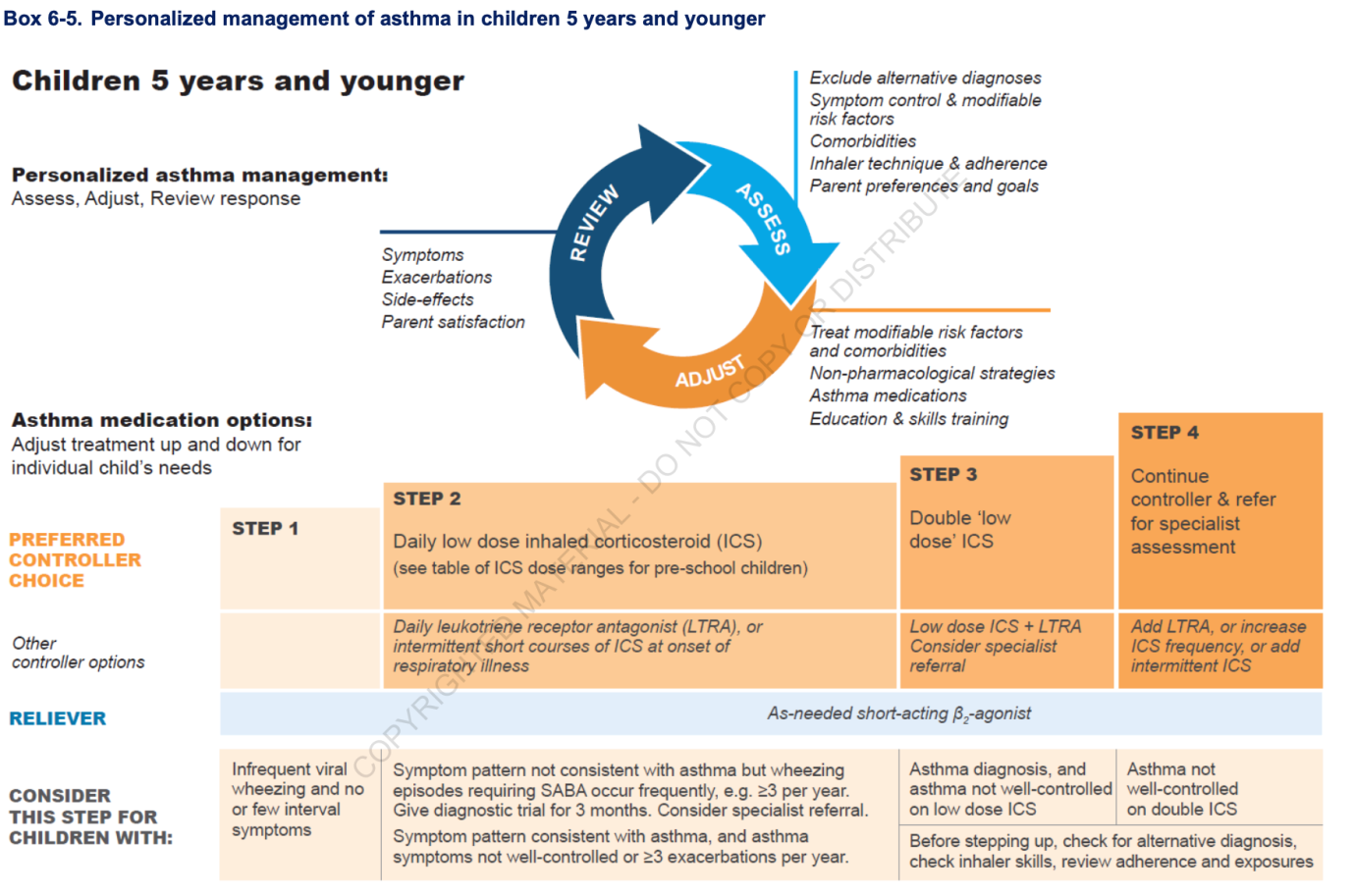


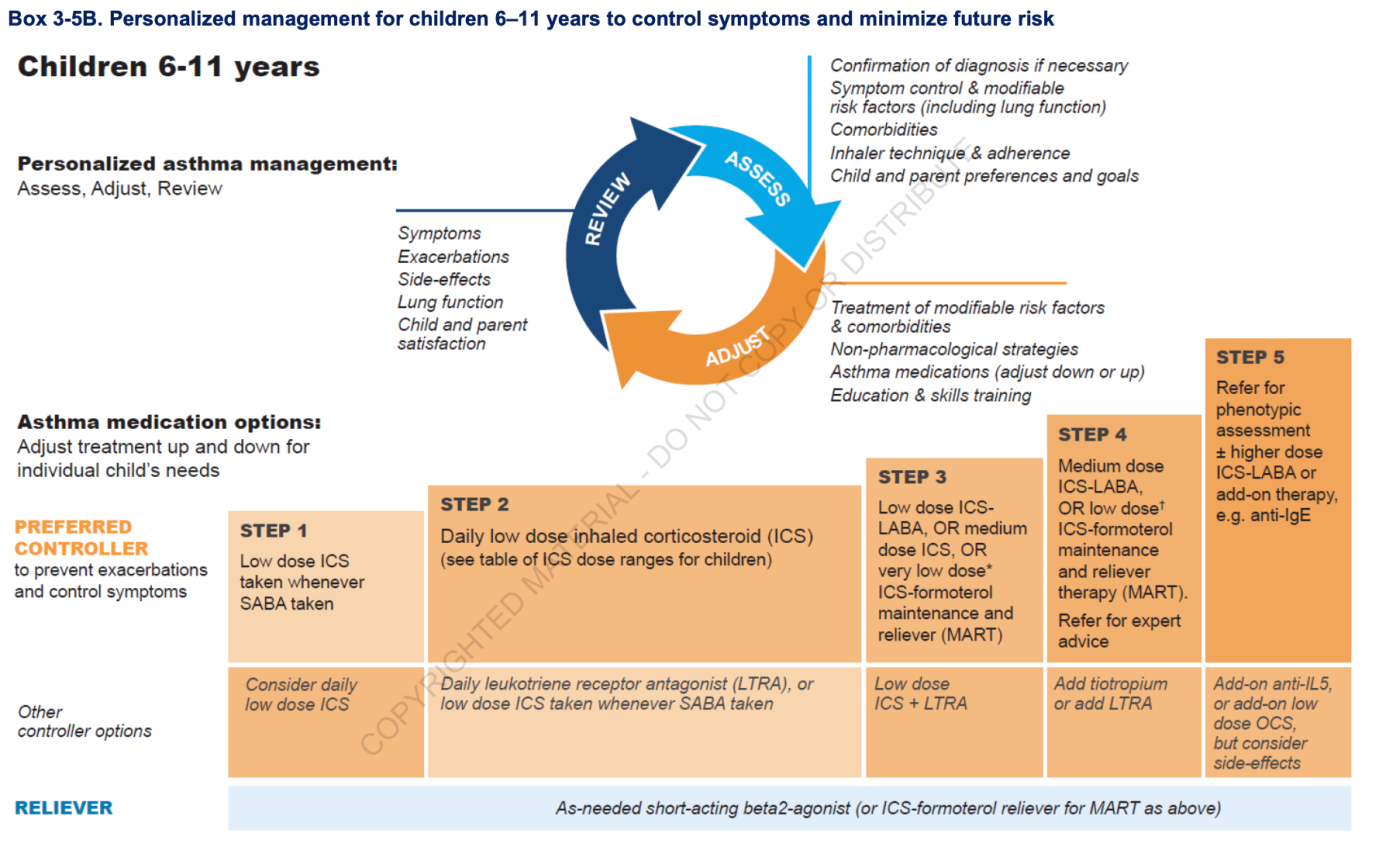


**Supplementary Fig. S2** GINA personalized management for CYP < 5 y, and 6–11 y

Reproduced, with permission, from: Global Initiative For Asthma. Global Strategy for Asthma Management and Prevention, 2021. Available from www.ginasthma.org


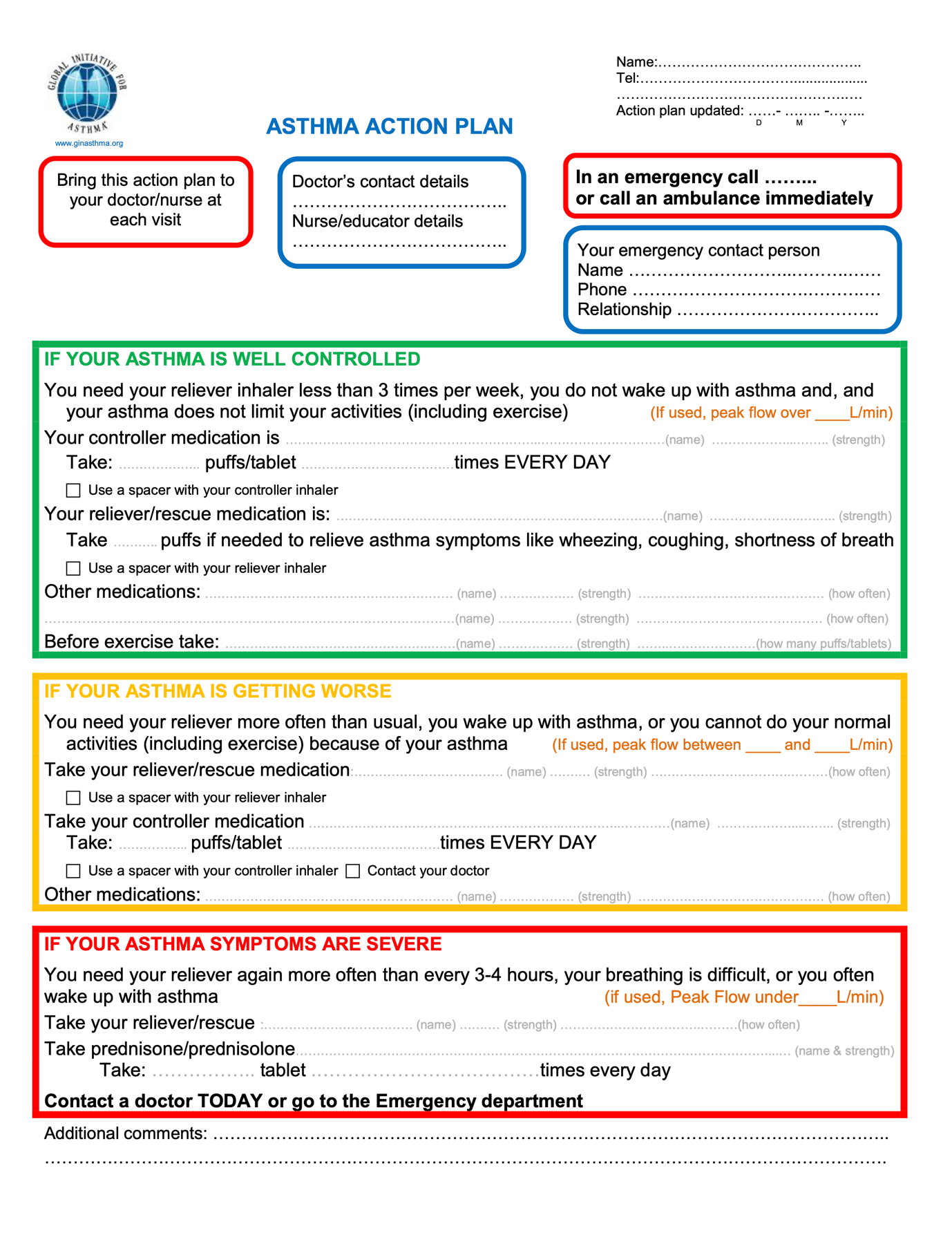


**Supplementary Fig. S3** GINA asthma action plan

Reproduced, with permission, from: Global Initiative For Asthma. GINA Implementation Toolbox, 2018. Available from www.ginasthma.org
